# Chemoselectivity in the Oxidation of Cycloalkenes with a Non-Heme Iron(IV)-Oxo-Chloride Complex: Epoxidation vs. Hydroxylation Selectivity

**DOI:** 10.1007/s13361-019-02251-1

**Published:** 2019-08-09

**Authors:** Thibault Terencio, Erik Andris, Ilaria Gamba, Martin Srnec, Miquel Costas, Jana Roithová

**Affiliations:** 1 Department of Organic Chemistry, Faculty of Science, Charles University, Hlavova 2030/8, 128 43 Prague 2, Czech Republic; 2 School of Chemical Science and Engineering, Yachay Tech University, 100650 Yachay City of Knowledge, Urcuqui Ecuador; 3 Departament de Quimica and Institute of Computational Chemistry and Catalysis (IQCC), University of Girona, Campus Montilivi, 17071 Girona, Spain; 4 J. Heyrovsky Institute of Physical Chemistry of the CAS, v. v. i., Dolejškova 2155/3, 182 23 Prague 8, Czech Republic; 5 Institute for Molecules and Materials, Radboud University Nijmegen, Heyendaalseweg 135, 6525 AJ Nijmegen, Netherlands

**Keywords:** C–H activation, DFT calculations, Epoxidation, Gas-phase reactions, Iron complexes

## Abstract

We report and analyze chemoselectivity in the gas phase reactions of cycloalkenes (cyclohexene, cycloheptene, *cis-*cyclooctene, 1,4-cyclohexadiene) with a non-heme iron(IV)-oxo complex [(PyTACN)Fe(O)(Cl)]^+^, which models the active species in iron-dependent halogenases. Unlike in the halogenases, we did not observe any chlorination of the substrate. However, we observed two other reaction pathways: allylic hydrogen atom transfer (HAT) and alkene epoxidation. The HAT is clearly preferred in the case of 1,4-cyclohexadiene, both pathways have comparable reaction rates in reaction with cyclohexene, and epoxidation is strongly favored in reactions with cycloheptene and *cis-*cyclooctene. This preference for epoxidation differs from the reactivity of iron(IV)-oxo complexes in the condensed phase, where HAT usually prevails. To understand the observed selectivity, we analyze effects of the substrate, spin state, and solvation. Our DFT and CASPT2 calculations suggest that all the reactions occur on the quintet potential energy surface. The DFT-calculated energies of the transition states for the epoxidation and hydroxylation pathways explain the observed chemoselectivity. The SMD implicit solvation model predicts the relative increase of the epoxidation barriers with solvent polarity, which explains the clear preference of HAT in the condensed phase.

**Electronic supplementary material:**

The online version of this article (10.1007/s13361-019-02251-1) contains supplementary material, which is available to authorized users.

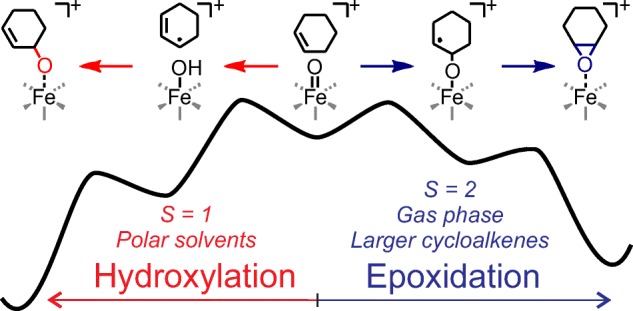

## Introduction

Iron(IV)-oxo compounds mediate diverse enzymatic oxidations including hydroxylation and halogenation of aliphatic C–H bonds, and epoxidation of alkenes and arenes [1–10]. Hallmark reactions among these are hydroxylations mediated by the iron(IV)-oxo porphyrin radical (Cpd I) of heme iron enzymes, such as cytochrome P450 [11–13], which received considerable attention in bioinorganic chemistry, leading to development of important mechanistic concepts, such as OH rebound [11, 14–16]. Since the beginning of this century, a lot of effort has been devoted to understanding oxidations mediated by non-heme systems [17–23]. Mechanistic studies of hydroxylation of aliphatic C–H bonds by α-ketoglutarate-dependent hydroxylase TauD [24], prolyl hydroxylase [25], and also of halogenation by non-heme iron-dependent halogenases [26] point toward an initial hydrogen atom abstraction by an iron(IV)-oxo species [27–30].

Oxidation of C–H and C=C bonds is a main topic in chemistry and specifically in catalysis research [31]. Even though high-valent iron systems excel at both of these transformations [32, 33], non-heme iron(IV)-oxo species generally prefer C–H over the C=C bond oxidation [34, 35]. The epoxidation has been achieved by additives such as acids [36], or using iodosylarene-based oxidants, instead of iron-oxo species [37] or using substrates with deuterated C–H bonds [38]. Notable exceptions include a macrocyclic iron(IV)-oxo described by Rybak-Akimova [39], and the [(cyclam)Fe(O)(CH_3_CN)]^2+^ system [40], where the C=C epoxidation preference has been attributed to hydrogen bonding of the cyclam ligand [41]. Additional factors that have been suggested to govern the C–H vs. C=C selectivity include spin inversion probability [38] and the spin state of the iron complex [41].

**Scheme 1:**
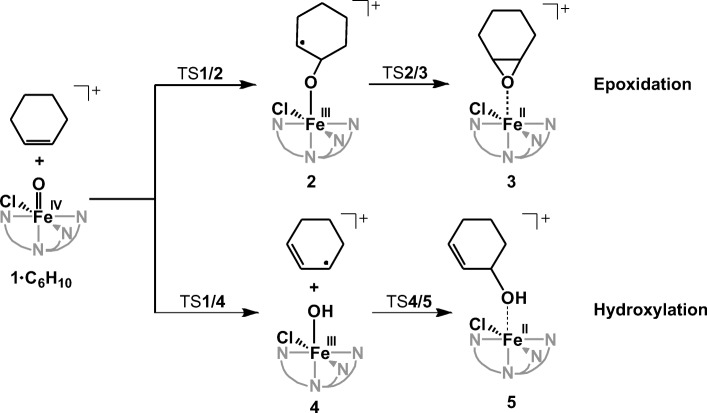
Oxidation of cyclohexene by iron(IV)-oxo complex **1** leading to epoxidation or hydroxylation. Alternatively, complex **4** can rebound the C-radical with the chlorine atom (not shown)

Epoxidation starts with the coordination of the oxygen atom of the iron complex to a carbon atom of the double bond of an alkene. The epoxide ring closes by the attack of the second carbon atom at the oxygen atom (Scheme 1). Hydroxylation starts by H atom abstraction leading to the allyl radical and iron(III)-hydroxo intermediate. In the second step, the allyl radical and the hydroxyl groups rebound to form allyl alcohol [28]. The rate-determining step in the hydroxylation reaction is HAT (hydrogen atom transfer). In agreement, Mayer et al. found that the rate constants for α-hydroxylation of alkenes by iron(IV)-oxo complexes linearly correlate with their C–H bond dissociation energies (BDEs) [42]. Such a correlation is commonly referred to as the Bell–Evans–Polanyi (BEP) relationship.

Previous computational studies dealing with selectivity between hydroxylation and epoxidation of propene show that environmental effects mimicking the protein pocket disadvantage epoxidation, whereas lower barriers toward epoxidation are observed in the gas phase [43]. De Visser et al. showed that the activation energy for epoxidation correlates linearly with the ionization energy of substrates [44]. Not surprisingly, the reactivity trends are further influenced by the nature of the metal complexes, especially the coordination sphere of the metal which plays the key role [45, 46].

Many aspects of these important oxidation reactions were already addressed [47–50], yet a debate continues. One persisting and challenging topic in this research area is the effect and the role of a spin state of iron(IV)-oxo complexes [49, 51–53]. It was numerously shown that reactivities of iron(IV)-oxo complexes in the high-spin state are higher than those of intermediate- or low-spin state complexes [54, 55], but exceptions also do exist [41, 56]. Other topics may include questions about the selectivity between C–H and C=C oxidation [57–59] or about solvent effects [60].

**Figure 1:**
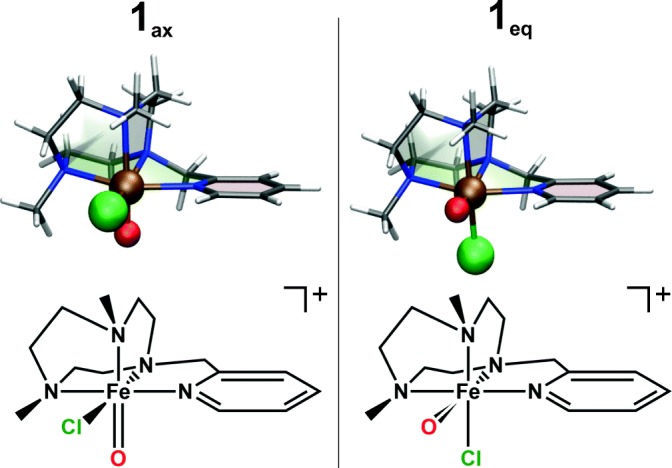
Structures of the two stereoisomers of the studied iron(IV)-oxo complex [(PyTACN)Fe(O)(Cl)]^+^

This paper addresses these questions by comparing experimental and theoretical data for non-heme iron(IV)-oxo complex [(PyTACN)Fe(O)(Cl)]^+^ (**1**, PyTACN = 1-[(2-pyridyl)methyl]-4,7-dimethyl-1,4,7-triazacyclononane; Figure 1) in oxidation of cyclohexene, cycloheptene, and *cis-*cyclooctene. The complex has been previously studied in the condensed phase (acetonitrile, 243 K) and it oxidized sulfides to sulfoxides and abstracted hydrogen atoms from weak C–H bonds [61]. The intended halogenation of substrates was not observed. Instead, a detailed isotopic labelling study with triphenylmethane suggested that the complex **4** might undergo hydroxyl group rebound exclusively.

## Materials and Methods

### Experimental Methods

Reactivity of the [(PyTACN)Fe(O)(Cl)]^+^ ions was investigated with a TSQ 7000 mass spectrometer with a quadrupole-octopole-quadrupole (Q1-O-Q2) configuration equipped with an electrospray ionization ion source (ESI) [62, 63]. Ions were generated by collisional activation of the [(PyTACN)Fe(NO_3_)(Cl)]^+^ precursors leading to the NO_2_^•^ loss in the ion source [64]. The precursor [(PyTACN)Fe(NO_3_)(Cl)]^+^ ions were transferred from an acetonitrile solution, which contained 100 μM of [(PyTACN)Fe(OTf)_2_] (for synthesis, see [65]), 50 μM of hydrogen peroxide to oxidize the Fe^II^ to Fe^III^, 200 μM of HNO_3_, and 32 μM of HCl. The stock solutions of the components were prepared by dilution of commercially available 65% nitric acid, 37% hydrochloric acid, and 30% hydrogen peroxide in acetonitrile. ESI source conditions were 6.5 kV spray voltage, 30 psi sheath gas pressure, − 20 V capillary voltage, 80 V tube lens voltage, 200 μl/h flow rate, and 120 °C capillary temperature.

The [(PyTACN)Fe(O)(Cl)]^+^ ions were mass selected by the first quadrupole and collided at nominally zero collision energy (Figure S1) with neutral reactants in the octopole collision cell. The products of the collisions were mass analyzed by the second quadrupole. Pressure of the neutral reactants in the collision cell was measured with 120 AA Baratron (MKS Instruments). Reaction cross sections *σ* were measured at three different pressures and the rate constants were extracted from the linear fits of the pressure dependence of *σ* with the *σ-*intercept set to 0 [64].

The 1,4-cyclohexadiene-(1,2,3,4,5,6)-*d*_6_ was prepared by a Birch reduction of benzene-*d*_6_ as described earlier [64].

### Theoretical Methods

Density functional theory (DFT) calculations were performed using the B3LYP-D3 method [66–69] implemented in Gaussian 09 (Gaussian 16) package [70, 71], which previously showed good agreement between the predicted potential energy surfaces (PESs) and the experimental data for similar systems [52, 72, 73]. Structures were optimized using 6-31G* basis set. The identity of the stationary states was verified by frequency calculations. For some structures, we were not able to eliminate a small imaginary frequency corresponding to a relative rotation of the iron complex and the cycloalkane substrate; these cases are indicated in the XYZ file with optimized structures. The charge and spin distributions were calculated using natural bond order (NBO) analysis [74]. The final energies were obtained as a sum of the single-point energy calculated with the 6-311++G** basis set and zero-point energy and thermal and entropic corrections calculated with unscaled double ζ vibrational frequencies. The solvation effect was studied by repeating the whole calculation process with a solvation model based on density (SMD) [75], where the gas-phase–optimized structures were used as the starting geometries. Note that specific interactions between solute and solvent molecules such as hydrogen bonding are not included in this solvation model.

The complete active space self-consistent field (CASSCF) [76] and complete active space second-order perturbation theory (CASPT2) [76–79] calculations were carried out using the MOLCAS 8.0 program [80]. For all of the atoms, the ANO-RCC basis set, contracted to [6s5p3d2f1g] for Fe, [4s3p2d] for the ligating O, N atoms, [3s2p] for C atoms, [4s3p] for Cl, and [2s] for H, was used. The second-order Douglas–Kroll–Hess (DKH2) one electron spinless Hamiltonian was applied for all of the calculations in order to allow for spin-free relativistic effects [81–83]. The CASSCF energies were calculated for the B3LYP-D3 optimized geometries with the 12-electrons-in-9-orbitals active space including 5x3d_Fe_, 3x2p_oxo_, and 1σ chelate-based orbital. To improve the accuracy of the calculations, the CASPT2 energies were used on the diagonal of the two-component Hamiltonian matrix. To approximate the two electron integrals, the Cholesky decomposition technique with a threshold of 10^−6^ au was used [84]. In all of the CASSCF calculations, a level shift of 5 au was used in order to improve convergence. In the CASPT2 calculations, none of the orbitals was frozen, and an imaginary level shift of 0.2*i* au was used to eliminate intruder states.

## Results

### Gas-Phase Reactivities

**Figure 2:**
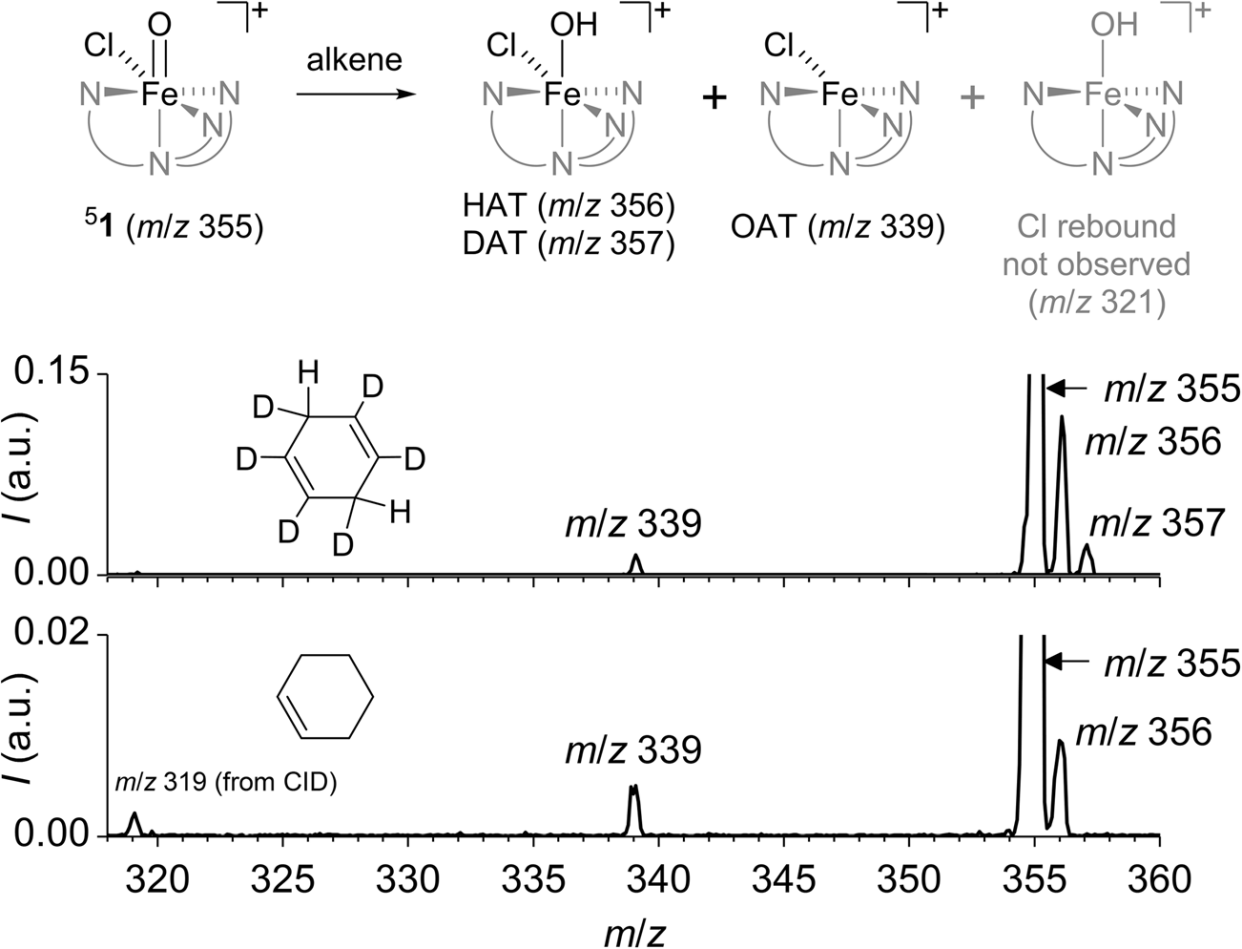
Product mass spectra of reaction between [(PyTACN)Fe(O)(Cl)]^+^ and 1,4-cyclohexadiene-1,2,3,4,5,6-*d*_6_ (the upper spectrum) or cyclohexene (the lower spectrum) at nominally zero collision energy. The spectra show a small contamination by the HCl elimination product at *m*/*z* 319, which results from collision-induced dissociation (CID) of [(PyTACN)Fe(O)(Cl)]^+^ (Figure S2)

The mass-selected [(PyTACN)Fe(O)(Cl)]^+^ ions (**1**, *m/z* 355) react with cycloalkenes (cyclohexene, cycloheptene, *cis-*cyclooctene, and 1,4-cyclohexadiene) by hydrogen atom transfer and oxygen atom transfer (HAT and OAT; Table 1). Because the [(PyTACN)Fe(O)(Cl)]^+^ complex contains *cis*-Fe^IV^(O)(Cl) motif present in non-heme iron-dependent halogenases, we hypothesized that the complex will react with the substrate via an initial hydrogen atom transfer followed by chlorine rebound. However, we have detected neither chlorine rebound nor chlorine atom transfer (Figure 2).

**Table 1:** Experimental Reaction Rates of HAT and OAT by [(PyTACN)Fe(O)(Cl)]^+^ in the Gas Phase

Alkene	*k*_HAT_ (10^−12^ cm^3^ s^−1^)	*k*_OAT_ (10^−12^ cm^3^ s^−1^)	KIE
Cyclohexene	0.62 ± 0.04	0.50 ± 0.12	–
Cycloheptene	0.16 ± 0.01	1.75 ± 0.10	–
*cis*-Cyclooctene	0.23 ± 0.01	1.85 ± 0.09	–
1,4-Cyclohexadiene	9.2 ± 0.8	0.38 ± 0.04	–
1,4-Cyclohexadiene-(1,2,3,4,5,6)-*d*_6_	5.1 ± 1.3^a^	0.51 ± 0.13	5.6 ± 0.2

^a^*k*_DAT_ = (0.90 ± 0.23) × 10^−12^ cm^3^ s^−1^

^b^The rate constant was normalized against the known rate constant for the reaction of [(PyTACN)Fe(O)(NO_3_)]^+^ cations generated by the nitrate cleavage with 1,4-cyclohexadiene-(1,2,3,4,5,6)-*d*_6_ ((13.9 ± 1.4) × 10^−12^ cm^3^ s^−1^) [64]

Instead, we observed exclusively products resulting from HAT and OAT (oxygen atom transfer). The ratios between the observed abundances of OAT and HAT depend on the size of the cycloalkene reactant. The reactions of larger cycloalkenes (cycloheptene and cyclooctene) with [(PyTACN)Fe(O)(Cl)]^+^ lead to OAT with rather high selectivity (about 90%). The reaction of cyclohexene is not selective with branching between HAT and OAT of about 55:45.

We also investigated the oxidation reaction with 1,4-cyclohexadiene having much more reactive allylic C-H bonds, because the HAT-generated radical is stabilized by π-electron delocalization. Accordingly, we observed selectivity toward HAT being more than 95%. We have compared this result with the very same reaction performed with 1,4-cyclohexadiene-(1,2,3,4,5,6)-*d*_6_. The substrate has both methylene groups half deuterated and therefore the comparison of HAT and DAT (deuterium atom transfer) gives directly the kinetic isotope effect (KIE). KIE amounts to 5.6, which is consistent with the previous analogous C–H activation reactions studied in the gas phase [73].

Gaseous C–H activation reaction can proceed within the reaction complex formed by HAT by a rebound step to form the alcohol product [2, 28]. In this case, the epoxidation channel would lead to the same signals in the MS spectrum as the HAT channel followed by the rebound step. Therefore, it was important to estimate the share of the rebound step. If the HAT + rebound pathway were to contribute to the overall observed OAT channel, then the rate observed for OAT would have to drop upon the deuterium labelling. In fact, we observed the opposite effect (see Table 1) which means that OAT corresponds dominantly to epoxidation. Competition between HAT and OAT explains the slight increase of the OAT rate upon deuterium labelling. Thus, we assumed that the possible contribution of the HAT + rebound to the OAT channel could be neglected. This assumption was further supported by our calculations of the OH and Cl rebound reactions in the next section, which showed that the Cl rebound channel proceeds over lower energy barriers. Thus, if the rebound reaction occurred, it should have been at least partially the Cl rebound, which was, however, not observed.

### DFT Calculations

### The [(PyTACN)Fe(O)(Cl)]^+^ Complex

The [(PyTACN)Fe(O)(Cl)]^+^ complex has two stereoisomeric structures shown in Figure 1 and denoted as **1**_ax_ and **1**_eq_, with the oxygen atom either in the axial or in the equatorial position, respectively [64]. We considered both isomers in the triplet and quintet states. Our DFT calculations as well as CASPT2 calculations suggest that both **1**_ax_ and **1**_eq_ are quintets and that ^5^**1**_ax_ is the most stable isomer (Table 2). Interestingly, the experimental ground state of the complex in acetonitrile solution is the triplet state [61]. In agreement, our DFT calculations using the SMD solvation model (Table 2) show that the triplet is indeed stabilized by the solvation. Geometric and electronic parameters show only minor differences between the stereoisomers with the same spin state. The *S =* 1 and *S =* 2 complexes differ in the bonding toward the PyTACN ligand. The Fe–N distances are shorter in the *S* = 1 complex than in the *S* = 2 complex; the Fe–O and Fe–Cl bonds are not affected.

**Table 2:** Energies in kJ mol^−1^ and Selected Interatomic Distances in Å for Axial and Equatorial Conformations of [(PyTACN)Fe(O)(Cl)]^+^ Complex, in the *S* = 2 and *S* = 1 Spin States

Conformation	Axial						Equatorial					
Spin state	Quintet (^5^**1**_ax_)			Triplet (^3^**1**_ax_)			Quintet (^5^**1**_eq_)			Triplet (^3^**1**_eq_)		
Solvent	Vacuum	DCM	H_2_O	Vacuum	DCM	H_2_O	Vacuum	DCM	H_2_O	Vacuum	DCM	H_2_O
E(DFT)^a^	0	0	0	13	8	3	3	0	− 4	9	3	− 2
E(CASPT2)^c^	0	–	–	10	–	–	5	–	–	41	–	–
Fe–O	1.61	1.62	1.63	1.62	1.63	1.63	1.61	1.62	1.62	1.62	1.62	1.63
Fe–N^d^	2.19	2.19	2.18	2.07	2.06	2.05	2.19	2.18	2.17	2.06	2.05	2.04
Fe–Cl	2.25	2.32	2.33	2.26	2.32	2.33	2.28	2.38	2.41	2.28	2.34	2.36

^a^B3LYP-D3/6-311++G**//B3LYP-D3/6-31G* energies at 0 K including ZPE correction relatively to ^5^**1**_ax_ (*S* = 2 spin isomer with the oxygen atom in the axial position)

^b^Geometries were optimized at the B3LYP-D3/6-31G* level of theory

^c^CASPT2 electronic energies

^d^Average of the four Fe–N distances

### Epoxidation and Hydroxylation of Cyclohexene

In the following, we will analyze reactivity of the iron(IV)-oxo complex on the quintet potential energy surface. All minima as well as transition structures discussed here have the *S* = 2 ground state; we analyze the effect of the spin state in the next subsection.

**Figure 3:**
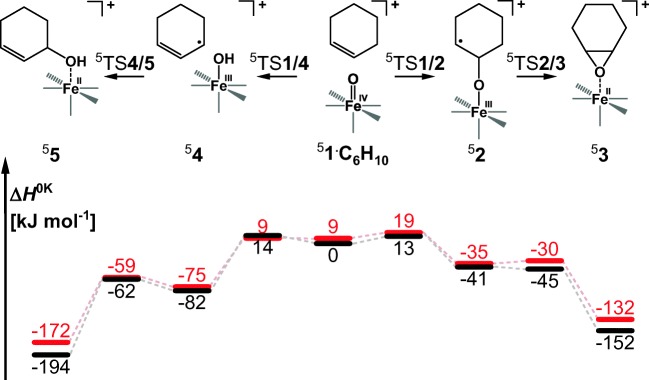
Potential energy surface (*S* = 2) for the reaction between cyclohexene and [(PyTACN)Fe(O)(Cl)]^+^ with the oxygen atom in the axial (black) or the equatorial (red) position in the gas phase. The energies are given at 0 K relative to ^5^**1**_ax_•C_6_H_10_ reactant complex. Relative enthalpies of the separated reactants for the axial and equatorial complexes are 36 and 40 kJ mol^−1^, respectively

The association between cyclohexene and complexes ^5^**1**_ax_ and ^5^**1**_eq_ is exothermic by 36 and 40 kJ mol^−1^, respectively. Selectivity between epoxidation and α-hydroxylation is determined by the first, rate-determining energy barrier that leads either to the O-atom addition to the C=C double bond or to the H-atom abstraction (transition structures ^5^TS**1**/**2** and ^5^TS**1**/**4** in Figure 3). These two competitive energy barriers starting from *S* = 2 **1**_**ax**_ are calculated to be 13 kJ mol^−1^ and 14 kJ mol^−1^, respectively (black profile in Figure 3). These similar barriers agree with roughly the same HAT and OAT reaction rates observed experimentally. The calculated intramolecular KIE for the ^5^TS**1**/**4** with cyclohex-1-ene-3-d is 3.2, which is slightly smaller than the KIE observed in reaction with 1,4-cyclohexadiene (5.6).

The epoxidation reaction leads via the radical intermediate ^5^**2**, which collapses to form the epoxide product ^5^**3** in a barrierless process. Overall, this results in a highly exothermic formation of the epoxide product (− 152 kJ mol^−1^).

The alternative hydroxylation pathway leads through a loosely bound intermediate between the iron(III) hydroxo complex and an allyl radical (^5^**4**). This complex can make an OH rebound to form the 3-hydroxycyclohexene via an energy barrier of ~20 kJ mol^−1^ (^5^TS**4**/**5**) or a Cl rebound via an energy barrier of 13 kJ mol^−1^. The formation of the hydroxyalkene is thermodynamically favored over epoxidation by 40 kJ mol^−1^. On the other hand, the formation of chlorinated product is disfavored by ~50 kJ over the epoxidation. In the gas phase, the allyl radical escapes from intermediate ^5^**4** and we observe formation of [(PyTACN)Fe(OH)(Cl)]^+^. Solvent molecules dissipate the energy released in the HAT process, thereby prolonging the lifetime of ^5^**4**, and thus facilitating the rebound step.

### Effect of the Spin State

**Figure 4:**
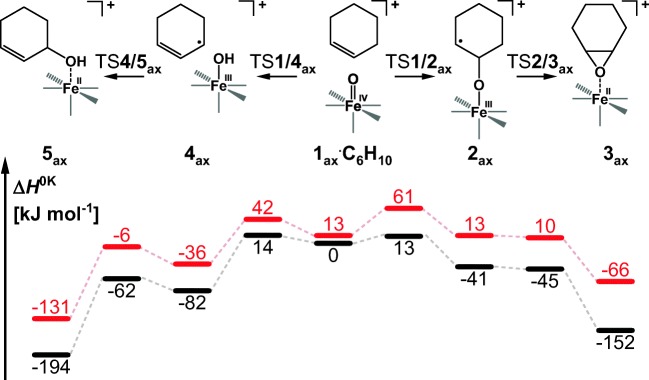
Potential energy surface for the reaction between cyclohexene and [(PyTACN)Fe(O)(Cl)]^+^ with the oxygen atom in the axial position in the *S* = 2 (black) and *S* = 1 (red) spin states. The energies are given at 0 K relative to the energy of ^5^**1**_ax_•C_6_H_10_ reactant complex. Relative enthalpies of the separated reactants for the quintet and triplet complexes are 36 and 50 kJ mol^−1^, respectively

The *S =* 1 potential energy surface lies higher in energy along all investigated reaction pathways (Figure 4). The high-spin iron complexes are usually considered as more reactive, and if the ground state of the reactant complex is *S* = 1, then the reaction pathways are associated with two-state reactivity [85–87]. If we look at the *S* = 1 energy barriers for initial OAT (48 kJ mol^−1^) and HAT (29 kJ mol^−1^), then the HAT process is clearly preferred on the *S* = 1 potential energy surface. Nevertheless, the overall heights of the barriers suggest that this spin state plays negligible role in the reaction.

**Figure 5:**
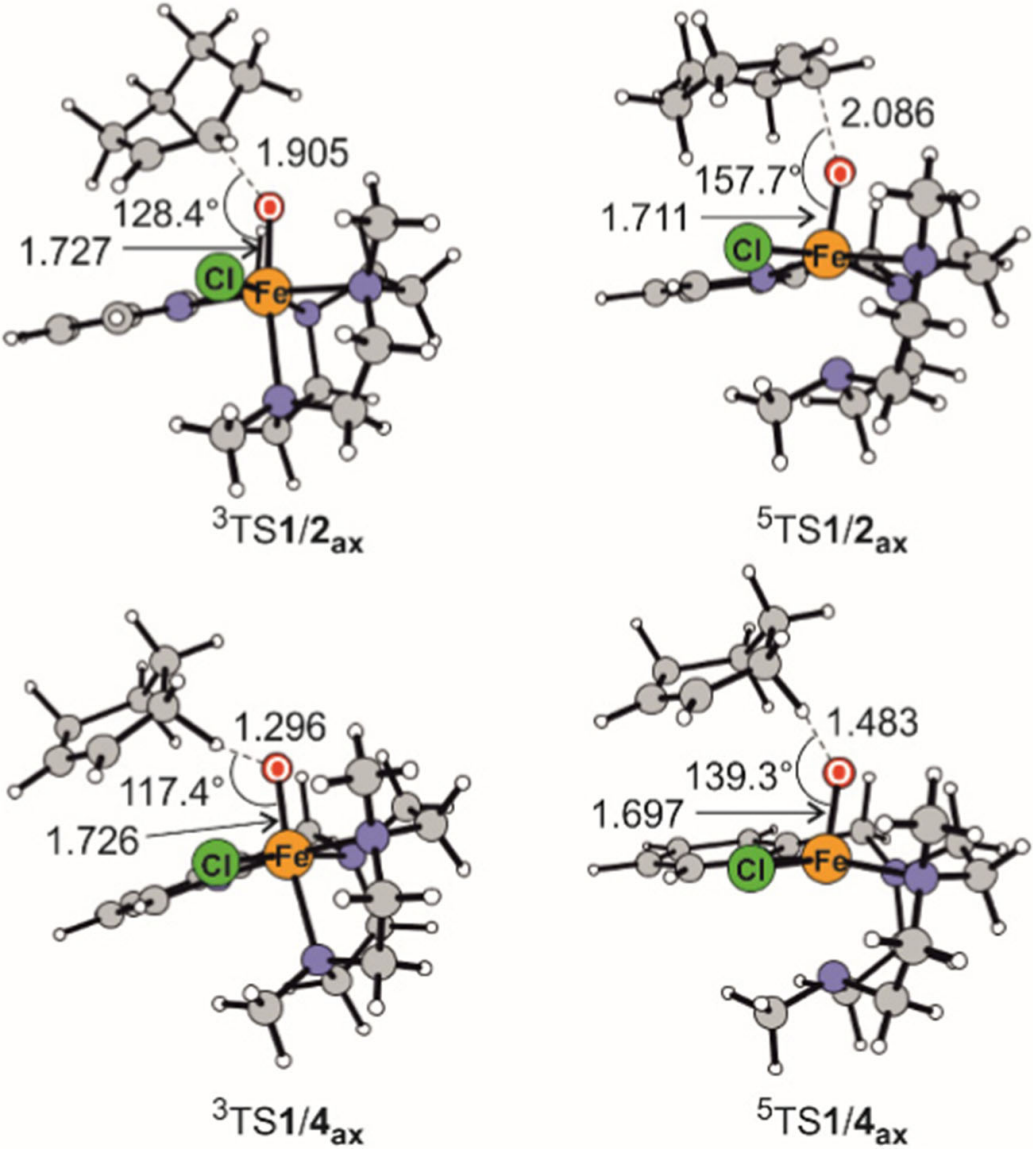
Optimized geometries of ^3^TS**1**/**2**_ax_, ^5^TS**1**/**2**_ax_, ^3^TS**1**/**4**_ax_, and ^5^TS**1**/**4**_ax_. The distances are given in ångströms

The large energy differences between the *S* = 1 vs. *S* = 2 transition states can be traced back to the geometries of the transition structures (Fig. 5). The epoxidation transition structures can be characterized by the Fe–O–C angle and the O–C distance. These parameters substantially differ for the *S* = 1 vs. *S* = 2 surface: 128.4° and 1.905 Å for ^3^TS**1**/**2**_ax_ vs. 157.7° and 2.086 Å for ^5^TS**1**/**2**_ax_. For the hydroxylation pathway, the rate-determining TS can be characterized by the Fe–O–H angle, the Fe–O and O–H distances. The values are 117.4°, 1.726 Å, and 1.296 Å for ^3^TS**1**/**4**_ax_ and 139.3°, 1.697 Å, and 1.483 Å for ^5^TS**1**/**4**_ax_, respectively.

Transition structures for both hydroxylation and epoxidation reaction pathways on the *S* = 1 potential energy surface can be characterized as π-like trajectories that require a closer approach of the substrate and smaller interaction angles [47]. On the contrary, the pathways on the *S* = 2 potential energy surface proceed via transition structures indicating σ trajectories. A larger tightness of the *S* = 1 TSs, which correlates with the higher barriers on the triplet potential energy surfaces, is also reflected by the imaginary frequencies associated with them: 1624*i* cm^−1^ for ^3^TS**1**/**4**_ax_ vs. 877*i* cm^−1^ for ^5^TS**1**/**4**_ax_. Similarly, the epoxidation pathway is characterized by 416*i* cm^−1^ for ^3^TS**1**/**2**_ax_ and 178*i* cm^−1^ for ^5^TS**1**/**2**_ax_.

### Effect of the Oxygen Position in the Iron(IV)-Oxo Complex

For the equatorial isomer, the initial reactant complex lies at about the same energy as the transition structure for HAT. The transition structure for OAT is 10 kJ mol^−1^ higher in energy (Fig. 3). Therefore, epoxidation by the equatorial isomer should be disfavored. Experimentally, we have probably sampled a mixture of the axial and equatorial isomers because of the small energy difference in their stability (^5^**1**_ax_ is 3 kJ mol^−1^ more stable than ^5^**1**_eq_). The slight preference for HAT over OAT (i.e., 1.2:1 branching ratio in favor of hydroxylation observed in the experiment) is therefore consistent with the theoretical results (cf. Table 1).

### Effect of Solvent (SMD Model)

The effect of the solvent was investigated employing a continuum polarization method SMD for dichloromethane and water (direct interactions of the solvent molecules, like hydrogen bonding, are not taken into account in these calculations). The results show that the energy barriers for both, epoxidation and hydroxylation, increase with the increasing polarity of the solvent. This effect is more pronounced for the epoxidation pathway (Table 3).

**Table 3:** Relative Enthalpies at 0 K and Gibbs Energies at 298 K Along the Hydroxylation and the Epoxidation Pathways in Reaction of ^5^**1**_ax_ with Different Substrates in Different Solvents

			Hydroxylation				Reactants		Epoxidation			
	Reactant	Solvent	**5**	TS**4/5** (OH reb.)	**4**	TS**1/4** (HAT)	**1•**alkene	**1** + alkene^b^	TS**1/2**	**2**	TS**2/3**	**3**
Δ*H*^0K^ (kJ mol^−1^)	C_6_H_10_	None	− 194	− 62	− 82	14	0	36	13	− 41	− 45	− 152
	C_7_H_12_	None	− 191	− 63	− 84	13	0	34	5	− 62	− 66	− 166
	*cis*-C_8_H_14_	None	− 202	− 57	− 72	12	0	36	11	− 56	− 59	− 166
Δ*G*^298K^ (kJ mol^−1^)	C_6_H_10_	None	− 192	− 60	− 81	16	0	− 6	16	− 35	− 39	− 146
	C_7_H_12_	None	− 184	− 56	− 76	19	0	− 2	16	− 49	− 45	− 151
	*cis*-C_8_H_14_	None	− 197	− 51	− 70	15	0	− 7	13	− 53	− 46	− 163
	C_6_H_10_	None	− 192	− 60	− 81	16	0	− 6	16	− 35	− 39	− 146
	C_6_H_10_	DCM	− 180	− 38	− 67	24	0	− 24	36	− 11	− 17	− 122
	C_6_H_10_	H_2_O	− 170	− 33	− 67	26	0	− 20	47	− 8	− 13	− 120

B3LYP-D3/6-311++G**//B3LYP-D3/6-31G* energies relative to reactant complex ^5^**1**_ax_•alkene (alkenes: C_6_H_10_ = cyclohexene, C_7_H_12_ = cycloheptene, or *cis*-C_8_H_14_ = *cis*-cyclooctene)

^a^Isolated reactants

The effect of solvation is most probably related to the stabilization of the charged-localized structures. The more localized the charge, the larger stabilization by polar solvents is expected. This relation implies that the separated reactants and products will be more stabilized by polar environment than the reaction complexes and transition structures along the reaction pathway because the charge is localized only at one reactant/product and not over the whole reaction complex. This implies that the relative energies of the intermediates and transition structures increase with respect to the reactants and products. This is fully consistent with our results. They show that energy barriers for both reaction pathways rise, if the reaction proceeds in solvent. It is also consistent with the fact that solvation disfavors epoxidation relative to hydroxylation because of a larger charge delocalization to the alkene reactant associated with the OAT step.

We have further compared the effect of solvents on the rebound reaction with Cl and OH. The Cl rebound reaction was not observed in solution [61] and we did not detect any rebound reaction in the gas phase. Our calculations show that the OH rebound is strongly thermodynamically favored over the Cl rebound in all solvents (Figure S3). On the contrary, the barrier for the Cl rebound is lower than that for the OH rebound. Hence, the kinetically preferred Cl rebound should occur in the gas phase if a rebound reaction would occur at all. In solution, many effects can play a role. For example, formation of relatively stable intermediates after the hydrogen transfer opens a possibility for a cage escape mechanism [88, 89].

### Effect of the Substrate Ring Size

Theoretical investigation of the reactivity of cycloalkenes can be complicated because of possible conformations of the larger rings. The most stable conformation of cyclohexene is a half-chair (Figure S4). Several stable conformations were found for the larger cycloalkenes, and in agreement with literature, the most stable ones were the chair for cycloheptene and the conformation with four atoms in the plane for *cis-*cyclooctene [90–95].

Energies along the reaction pathways toward epoxidation and hydroxylation in the gas phase are listed in Table 3. The epoxidation pathways for the cycloheptene and *cis*-cyclooctene substrates are associated with smaller initial energy barriers than their hydroxylation pathways, which corroborates the experimentally observed preference for epoxidations. However, the energy differences are somewhat smaller than expected. The reason may stem from the overestimated zero-point energy for the epoxidation transition structure (see discussion in the Supporting Information, Table S2 and Figure S5).

The energy barriers for HAT often correlate with the corresponding BDEs of the C–H bond (the BEP principle) [42]. The BDEs of the α-C–H bonds rise in a row from cyclohexene to *cis-*cyclooctene [96]. The corresponding TS**1/4** energy rises from cyclohexene to cycloheptene, but then drops for *cis-*cyclooctene. Hence, if we consider barrier heights in our fully optimized reaction pathways, we do not observe the correlation with BDEs. The reason may stem from other than electronic effects. On one hand, the larger cycloalkenes can experience a large steric hindrance in the approach to the iron-oxo complex; on the other, they can relax to a more favorable conformation and can thus adopt a more favorable transition-structure geometry (see Table S3). The relaxation of geometry is probably the reason why we do not observe the expected correlation. We note that the differences in BDEs and TSs energies are small; therefore, already small secondary effects can ruin the linear relationships.

Epoxidation pathway is often related with the energy required to remove an electron from the C=C double bond. Hence, the ionization energies of the studied cycloalkenes [97] should correlate with the calculated energy barriers associated with TS**1**/**2**. The ionization energies decrease from cyclohexene (IE = 8.94 eV) to cycloheptene (IE = 8.87 eV) and to *cis-*cyclooctene (IE = 8.82 eV). Again, the differences in IEs are small and do not correlate with the observed reactivity.

## Discussion

Our experimental and computational survey of epoxidation and hydroxylation pathways in reaction of an iron(IV)-oxo complex with different cycloalkene reactants suggests several factors how to affect selectivity of these reactions. In points, selectivity toward hydroxylation or epoxidation can be affected:

1. By substrate: According to the previous studies, cycloalkenes with low ionization energies will prefer epoxidation, while those with weak C–H bonds will prefer hydroxylation. However, we observed that the selectivity can be also influenced by steric effects as demonstrated here: constrained approach in the course of the C–H activation pathway may lead to the preference of the alternative epoxidation pathway.
2. By spin state of iron(IV)-oxo complex: Comparison of the *S* = 1 and *S* = 2 potential energy surfaces revealed that in the initial *S* = 1 barrier for epoxidation is much higher than that for hydroxylation, whereas the barriers were about the same in the *S* = 2 state. Hence, the *S* = 1 complexes might tend to favor hydroxylation over epoxidation much more than the *S* = 2 complexes.
3. By the geometry of iron(IV)-oxo complex: The hydroxylation path is not significantly influenced by the position of the oxygen atom in the iron(IV)-oxo complex (i.e., axial vs. equatorial). Epoxidation is disfavored in the equatorial configuration. The approach of the alkene to the oxygen atom in the equatorial position is sterically hindered by vicinity of several hydrogen atoms. The effect is much larger when the oxygen atom interacts with the carbon atom of the C–C double bond than in the abstraction of a hydrogen atom from the alkene.
4. By solvent: Polar media disfavor epoxidation over hydroxylation. Charge delocalization is larger along the epoxidation pathway and therefore transition structures and intermediates are less stabilized by solvation than the initial reactants. Hydroxylation reaction is more of a radical character and therefore changes in solvation along the reaction pathway are smaller [98].

## Conclusion

We report systematic investigation of reactions between [(PyTACN)Fe(O)(Cl)]^+^ and three different cycloalkenes (cyclohexene, cycloheptene, and *cis-*cyclooctene). Experimentally, either hydrogen atom or oxygen atom transfer occurred with different selectivities. The calculated potential energy surfaces rationalized why low or no selectivity was observed for reaction between the iron(IV)-oxo complex with cyclohexene, whereas the reactions with cycloheptene and cyclooctene showed large selectivity for epoxidation. Solvation largely affects the selectivity in favor of C–H activation. The epoxidation pathway is disfavored because of a larger charge delocalization in the key transition structure leading to a smaller stabilization by solvation.

Finally, our results suggest a strategy for the design of iron(IV)-oxo complex that would be selective for hydroxylation over epoxidation. It should be based on an equatorial configuration of the iron-oxo complex and the *S* = 1 spin state. The reactions should run in polar solvents. The other way around, the opposite strategy is predicted for enhancing selectivity of iron(IV)-oxo complexes toward epoxidation.

## Electronic Supplementary Material

ESM 1 (PDF 711 kb)

ESM 2 (XYZ 180 kb)

## Abbreviations

BDE: bond dissociation energy
BEP: Bell–Evans–Polanyi
DFT: density functional theory
HAT: hydrogen atom transfer
IE: ionization energy
OAT: oxygen atom transfer
