# Supplementary material for: Chemoselectivity in the Oxidation of Cycloalkenes with a Non-Heme Iron(IV)-Oxo-Chloride Complex: Epoxidation vs. Hydroxylation Selectivity
Source: J Am Soc Mass Spectrom. 2019 Aug 9;30(10):1923–33. doi: 10.1007/s13361-019-02251-1 (PMC6805805; doi:10.1007/s13361-019-02251-1)
Supplement: Supplementary file 1 — (PDF 711 kb) [file 13361_2019_2251_MOESM1_ESM.pdf]

Supporting Information for:

Chemoselectivity in the oxidation of cycloalkenes with a non-heme iron(IV)-oxo-chloride complex: Epoxidation vs. hydroxylation selectivity

Thibault Terencio,[1,2#] Erik Andris,[1#] Ilaria Gamba,[3] Martin Srnec,[4*] Miquel Costas,[3*] and Jana Roithová[1,51*]

[1]Department of Organic Chemistry, Faculty of Science, Charles University, Hlavova 2030/8, 128 43 Prague 2, Czech Republic.
[2]School of Chemical Science and Engineering, Yachay Tech University, Yachay City of Knowledge, 100650-Urcuqui, Ecuador.
[3]Departament de Quimica and Institute of Computational Chemistry and Catalysis (IQCC), University of Girona, Campus Montilivi, Girona 17071, Spain.
[4]J. Heyrovsky Institute of Physical Chemistry of the CAS, v. v. i., Dolejškova 2155/3, 182 23 Prague 8, Czech Republic.
[5]Institute for Molecules and Materials, Radboud University Nijmegen, Heyendaalseweg 135, 6525 AJ, Nijmegen, Netherlands.

[#] These authors contributed equally to this work.

[1] Correspondence to: Jana Roithova; jana.roithova@ru.nl; Miquel Costas; miquel.costas@udg.edu; Martin Srnec; martin.srnec@jh-inst.cas.cz.

Experimental measurements

Reaction rates were measured at nominally zero-collision energy (Figure S1) and at three different pressures. The slope of the reaction cross section plotted against the pressure (which is forced to go through the origin of the axes) gives the rate constant.

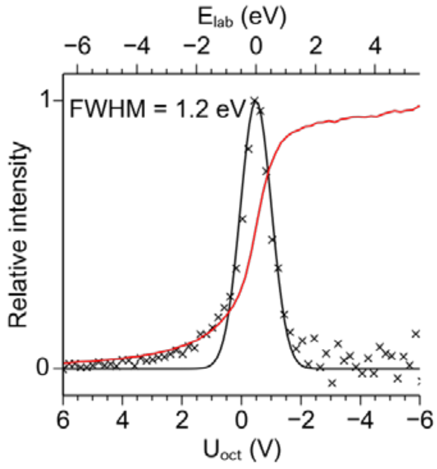

Figure S1. Typical ion kinetic energy distribution measured by the stopping potential analysis. The red line corresponds to the number of ions passing the octopole collision cell in dependence of the octopole potential offset. The black crosses correspond to the derivative of the red curve; the black line is a Gaussian fit of the experimental derivative.

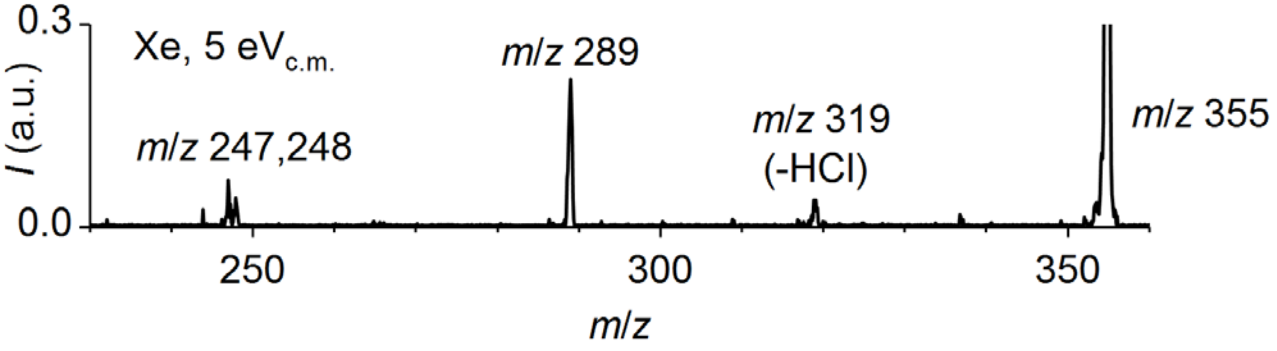

Figure S2. Collision induced dissociation spectrum of $[(PyTACN)Fe(O)(Cl)]^+$ ions with xenon at 5 eV (center-of-mass frame) collision energy.

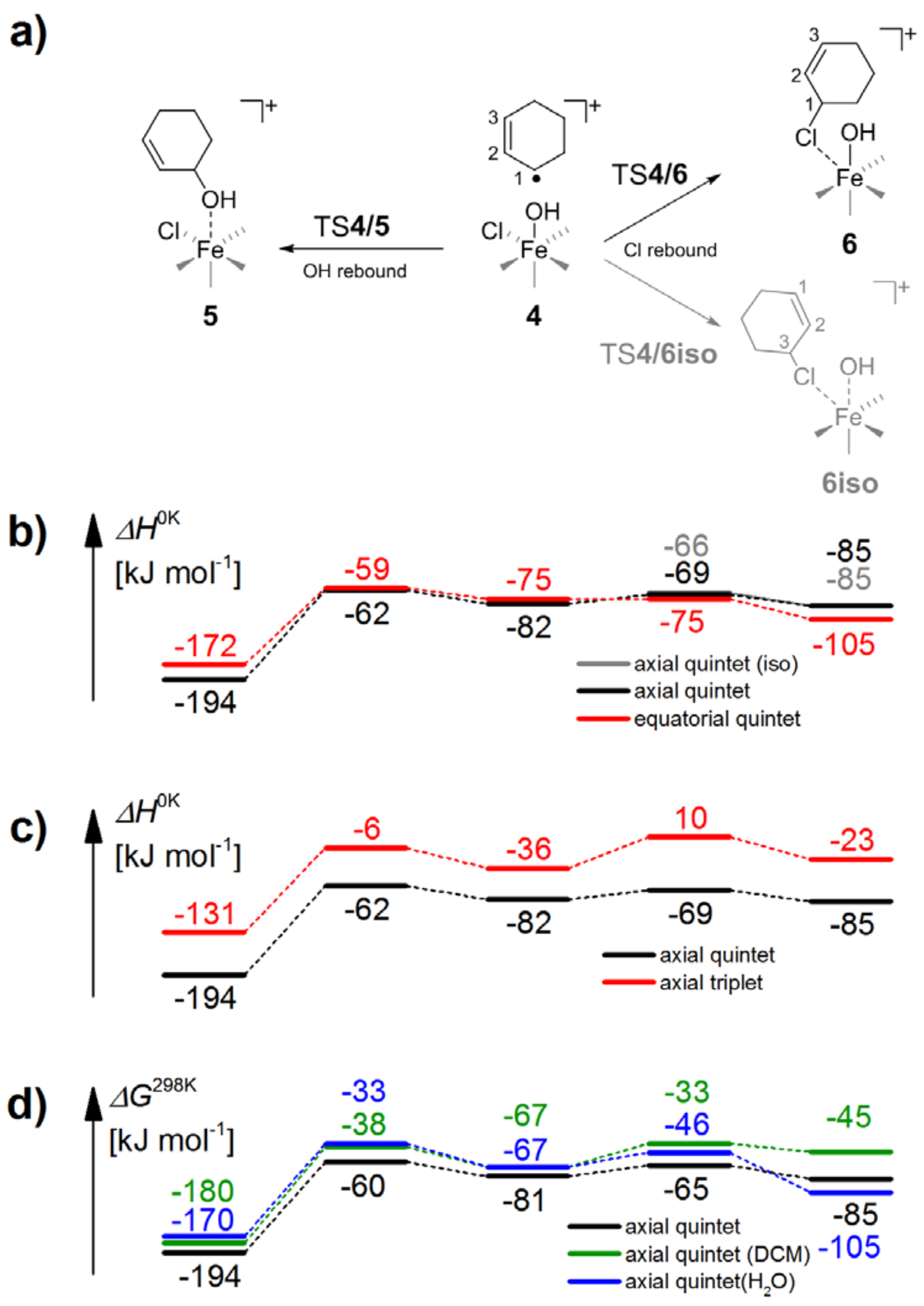

Figure S3. (a) Allylic radical produced in the HAT reaction of cyclohexene with $[(PyTACN)Fe(O)(Cl)]^+$ can undergo either OH rebound (left pathway) or chlorine rebound (right pathway). Because the allylic radical is localized on two carbons (labeled 1 and 3) and the carbon 3 is closer to the chlorine atom, the rebound can occur at both of these positions, leading to products 6 and 6iso, respectively. (b) Potential energy profiles for the OH/Cl rebound reactions in different isomers of $[(PyTACN)Fe(O)(Cl)]^+$ complex: (b) axial/equatorial quintet and (c) axial triplet/quintet. (d) Effect of the solvation on relative height of rebound channels (black lines = no solvation, green line = DCM, blue line = water).

Cycloalkenes conformations

Conformations were searched manually by arbitrary modification of the cycloalkenes. In this process, the literature was a fruitful source of inspiration.[1–6] For cyclohexene, only the "half-chair" conformation was located. For cycloheptene, four different conformations were found. The most stable corresponds to a "chair" conformation whereas the less stable one corresponds to a "boat" conformation. For cyclooctene, four different minima were also identified.

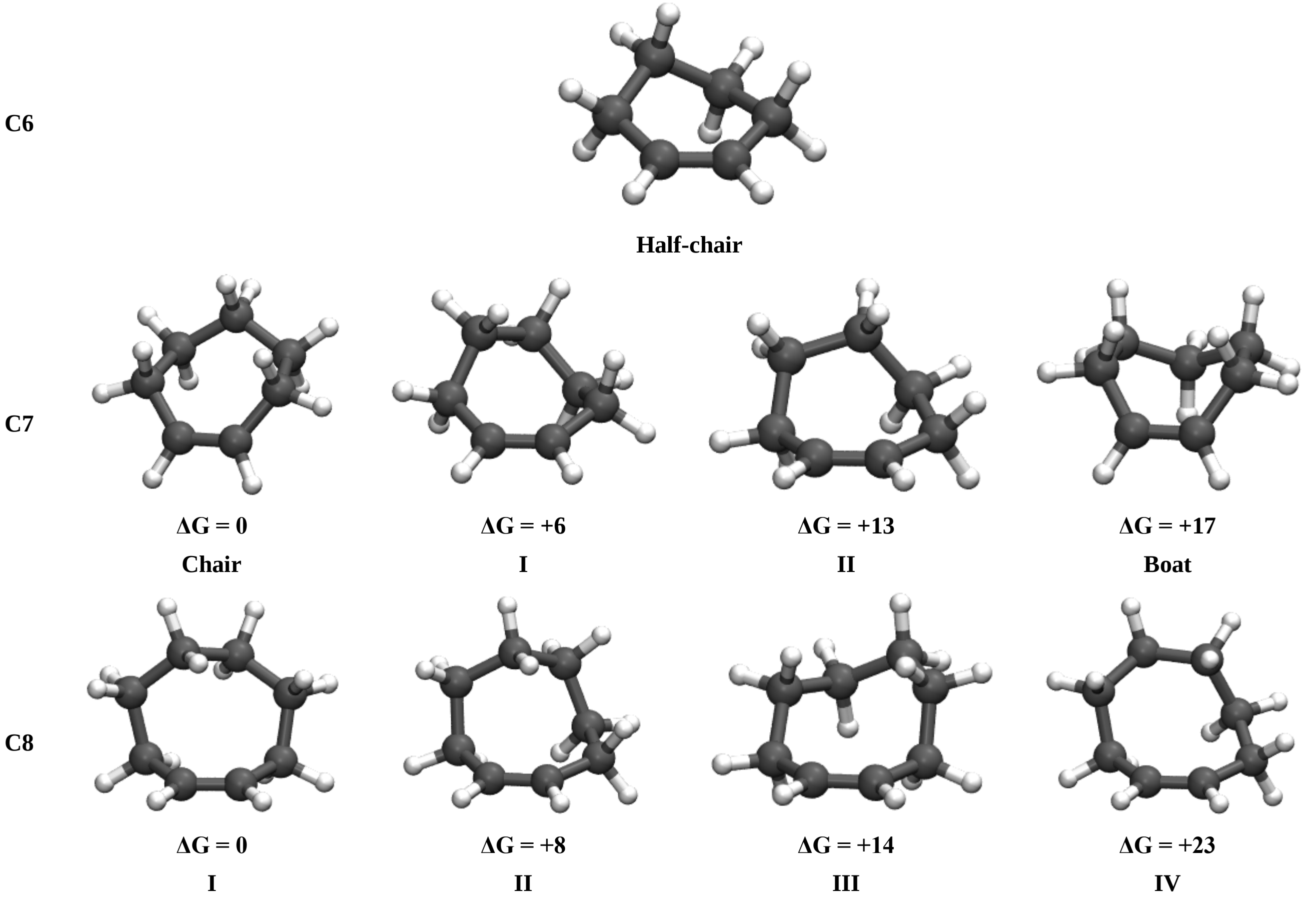

Figure S4. Stables conformations identified in this work for cyclohexene, cycloheptene and cyclooctene. Geometries and free energy differences were calculated using 6-311++G** basis-set and B3LYP-GD3BJ method.

Zero-point vibration energy contributions to TS1/2 and TS1/4

The energy difference between the transition structures **TS1/2** and **TS1/4** for reactions of cycloheptene and *cis*-cyclooctene are smaller than it would be expected based on the clear preference of these substrates for epoxidation. The electronic energy differences and differences of energies with zero-point vibrational energy (ZPVE) corrections and thermal corrections suggest that the zero-point vibrational energy (ZPVE) is overestimated. The electronic energy differences between **TS1/2** and **TS1/4** calculated with the 6-31G* basis set are -3, -14, -9 kJ.mol^{-1} for C6, C7, and C8 reactants, respectively. Single-point calculations with the triple ζ basis-set (6-311++G**) lead to similar results. The differences diminish to the values in Table 3 after the ZPVE correction (see Table S1 and Figure S5).

Table S2. Decomposition of the energetic components along the pathways for cyclohexene, cycloheptene and cyclooctene.

C6	Min 5	TS 4/5	Min 4	TS 1/4	Min 1	TS 1/2	Min 2	TS 2/3	Min 3
E (631Gd)	-2800.707654	-2800.656896	-2800.66809	-2800.635685	-2800.651158	-2800.641518	-2800.663505	-2800.662896	-2800.702736
E (SP BIG)	-2801.130384	-2801.075529	-2801.083328	-2801.04476	-2801.057448	-2801.049262	-2801.070471	-2801.071383	-2801.115259
ZPE	0.539138	0.53475	0.534673	0.53277	0.540182	0.536783	0.537678	0.537118	0.540222
G+ZPE	0.481299	0.476754	0.47625	0.474687	0.481343	0.479368	0.481061	0.480294	0.483711
G (631Gd)	-2800.226355	-2800.180142	-2800.19184	-2800.160998	-2800.169815	-2800.16215	-2800.182444	-2800.182602	-2800.219025
G(SP)	-2800.649085	-2800.598775	-2800.607078	-2800.570073	-2800.576105	-2800.569894	-2800.58941	-2800.591089	-2800.631548
ΔG (631Gd)	**-148**	**-27**	**-58**	**23**	**0**	**20**	**-33**	**-34**	**-129**
ΔG (SP)	**-192**	**-60**	**-81**	**16**	**0**	**16**	**-35**	**-39**	**-146**

C7	Min 5	TS 4/5	Min 4	TS 1/4	Min 1	TS 1/2	Min 2	TS 2/3	Min 3
E (631Gd)	-2840.019442	-2839.969765	-2839.98196	-2839.948765	-2839.963526	-2839.958085	-2839.985146	-2839.983722	-2840.021167
E (SP BIG)	-2840.452067	-2840.399141	-2840.407323	-2840.368467	-2840.380382	-2840.375787	-2840.401837	-2840.402948	-2840.443897
ZPE	0.567885	0.563914	0.564092	0.562042	0.569048	0.566262	0.566859	0.566453	0.569458
G+ZPE	0.507248	0.503291	0.503948	0.501213	0.505815	0.507327	0.508757	0.511086	0.511685
G (631Gd)	-2839.512194	-2839.466474	-2839.478012	-2839.447552	-2839.457711	-2839.450758	-2839.476389	-2839.472636	-2839.509482
G(SP)	-2839.944819	-2839.89585	-2839.903375	-2839.867254	-2839.874567	-2839.86846	-2839.89308	-2839.891862	-2839.932212
ΔG (631Gd)	**-143**	**-23**	**-53**	**27**	**0**	**18**	**-49**	**-39**	**-136**
ΔG (SP)	**-184**	**-56**	**-76**	**19**	**0**	**16**	**-49**	**-45**	**-151**

C8	Min 5	TS 4/5	Min 4	TS 1/4	Min 1	TS 1/2	Min 2	TS 2/3	Min 3
E (631Gd)	-2879.342164	-2879.284717	-2879.294621	-2879.264347	-2879.280137	-2879.272604	-2879.299323	-2879.298733	-2879.337168
E (SP BIG)	-2879.784592	-2879.724573	-2879.730436	-2879.695726	-2879.707676	-2879.701031	-2879.727093	-2879.728146	-2879.770784
ZPE	0.597974	0.593282	0.593266	0.590704	0.598074	0.595524	0.59606	0.59597	0.598107
G+ZPE	0.538608	0.534114	0.532835	0.530603	0.53674	0.535011	0.536154	0.539513	0.537753
G (631Gd)	-2878.803556	-2878.750603	-2878.761786	-2878.733744	-2878.743397	-2878.737593	-2878.763169	-2878.75922	-2878.799415
G(SP)	-2879.245984	-2879.190459	-2879.197601	-2879.165123	-2879.170936	-2879.16602	-2879.190939	-2879.188633	-2879.233031
ΔG (631Gd)	**-158**	**-19**	**-48**	**25**	**0**	**15**	**-52**	**-42**	**-147**
ΔG (SP)	**-197**	**-51**	**-70**	**15**	**0**	**13**	**-53**	**-46**	**-163**

The values in Table S1 show that the difference between the energies of TS1/2 and TS1/4 is bigger than the difference between their energies after the inclusion of ZPVEs. We plotted the ZPVE for each structure along both pathways. This data shows that ZPVE brings a bigger contribution to epoxidation than to hydroxylation, and especially in the case of the first transition states (TS1/2 and TS1/4). ZPVE corrections usually decrease with basis-set size. Consequently, adding ZPVE correction calculated at 6-31G* level to the single point 6-311++G calculations overestimates the ZPVE contribution to the energy. In other words, ZPVE correction increases effectively more the barrier towards epoxidation (TS1/2) than the barrier towards hydroxylation (TS1/4), decreasing the gap of energy between both transition states.**

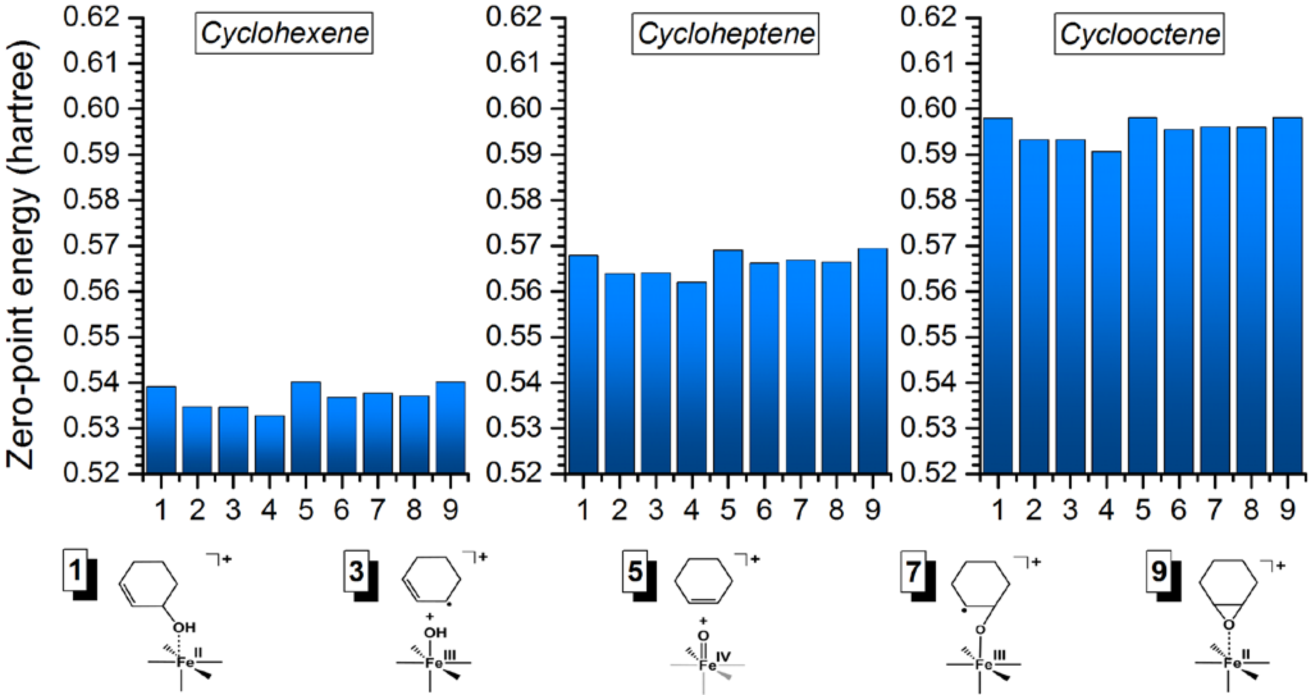

Figure S5. Zero point energies calculated for each structure along the hydoxylation and the epoxidation pathways for cyclohexene, cycloheptene and cyclooctene.

Key geometric parameters of transition structures TS1/2 and TS1/4. Distances are in Å and angles in degrees.

Table S3. Key geometrical parameters of transition structures TS1/2 and TS1/4.

reaction with C_6H_{10} in the gas phase								
	R(Fe-O)	R(O-C)	α(Fe-O-C)		R(Fe-O)	R(O-H)	R(C-H)	α(Fe-O-H)
$^5(TS1/2)_{ax}$	1.71	2.09	158	$^5(TS1/4)_{ax}$	1.70	1.43	1.19	139
$^5(TS1/2)_{eq}$	1.72	2.16	150	$^5(TS1/4)_{eq}$	1.69	1.39	1.21	156
$^3(TS1/2)_{ax}$	1.73	1.90	128	$^3(TS1/4)_{ax}$	1.73	1.30	1.28	117
reaction with C_6H_{10} in DCM								
$^5(TS1/2)_{ax}$	1.71	2.04	160	$^5(TS1/4)_{ax}$	1.71	1.30	1.26	169
reaction with C_6H_{10} in H_2O								
$^5(TS1/2)_{ax}$	1.73	1.97	159	$^5(TS1/4)_{ax}$	1.73	1.37	1.23	138
reaction with C_7H_{12} in the gas phase								
	R(Fe-O)	R(O-C)	α(Fe-O-C)		R(Fe-O)	R(O-H)	R(C-H)	α(Fe-O-H)
$^5(TS1/2)_{ax}$	1.68	2.27	165	$^5(TS1/4)_{ax}$	1.69	1.44	1.19	142
reaction with C_8H_{14} in the gas phase								
	R(Fe-O)	R(O-C)	α(Fe-O-C)		R(Fe-O)	R(O-H)	R(C-H)	α(Fe-O-H)
$^5(TS1/2)_{ax}$	1.70	2.22	157	$^5(TS1/4)_{ax}$	1.71	1.36	1.22	153

References

(1) Allinger, N. L. *J. Am. Chem. Soc.* **1977**, *99* (25), 8127–8134.

(2) Buemi, G.; Favini, G.; Zuccarello, F. *J. Mol. Struct.* **1970**, *5* (1–2), 101–110.

(3) Favini, G.; Buemi, G.; Raimondi, M. *J. Mol. Struct.* **1968**, *2* (2), 137–148.

(4) Jensen, F. R.; Bushweller, C. H. *J. Am. Chem. Soc.* **1969**, *91* (21), 5774–5782.

(5) Leong, M. K.; Mastryukov, V. S.; Boggs, J. E. *J. Mol. Struct.* **1998**, *445* (1–3), 149–160.

(6) Neuenschwander, U.; Hermans, I. *J. Org. Chem.* **2011**, *76* (24), 10236–10240.

(7) Wiberg, K. B.; Cheeseman, J. R.; Ochterski, J. W.; Frisch, M. J. *J. Am. Chem. Soc.* **1995**, *117* (24), 6535–6543.
